# In vivo evaluation of gray and white matter volume loss in the parkinsonian variant of multiple system atrophy using SPM8 plus DARTEL for VBM^☆^

**DOI:** 10.1016/j.nicl.2013.03.017

**Published:** 2013-04-05

**Authors:** Yoko Shigemoto, Hiroshi Matsuda, Kouhei Kamiya, Norihide Maikusa, Yasuhiro Nakata, Kimiteru Ito, Miho Ota, Naofumi Matsunaga, Noriko Sato

**Affiliations:** a Department of Radiology, National Center Hospital of Neurology and Psychiatry, 4-1-1 Ogawahigashi, Kodaira, Tokyo 187-8551 Japan; b Integrative Brain Imaging Center, National Center of Neurology and Psychiatry, 4-1-1 Ogawahigashi, Kodaira, Tokyo 187-8551 Japan; c Department of Mental Disorder Research, National Institute of Neuroscience, National Center of Neurology and Psychiatry, 4-1-1 Ogawa-Higashi, Kodaira, Tokyo 187-8502, Japan; d Department of Radiology, Yamaguchi University Graduate School of Medicine, 1-1-1 Minamikogushi, Ube, Yamaguchi 755-8505, Japan

**Keywords:** Multiple system atrophy with predominant parkinsonism (MSA-P), White matter, Diffeomorphic anatomical registration through exponentiated Lie algebra (DARTEL), Statistical parametric mapping (SPM), Voxel-based morphometry (VBM)

## Abstract

In multiple system atrophy with predominant parkinsonism (MSA-P), several voxel-based morphometry (VBM) studies have revealed gray matter loss; however, the white matter volume changes have been rarely reported. We investigated the volume changes of white matter as well as gray matter by VBM. A retrospective MRI study was performed in 20 patients with MSA-P and 30 age-matched healthy controls. We applied VBM with statistical parametric mapping (SPM8) plus diffeomorphic anatomical registration through exponentiated Lie algebra (DARTEL) to explore the regional atrophy of gray and white matter in all of the MSA-P patients, 14 patients with left-side dominant and 6 patients with right-side dominant onset as compared to controls. In all of the MSA-P patients, VBM revealed a significant volume reduction of gray matter in the bilateral putamina, cerebellums and dorsal midbrain. White matter loss was located in bilateral globus pallidi, external capsules extending to the midbrain, right subcortical to precentral area through internal capsule, the pons, bilateral middle cerebellar peduncles and left cerebellum. In left-side dominant MSA-P patients, the gray and white matter volume loss was detected predominantly on the right side and vice versa in right-side dominant MSA-P patients. A correlation with disease duration and severity was not detected. VBM using SPM8 plus DARTEL detected significant volume loss not only in the gray but also in the white matter of the area affected by MSA-P.

## Highlights

• Volume changes of gray and white matter were investigated by VBM in MSA-P.
• Volume loss of globus pallidus with each structure was detected.
• Gray and white matter volume loss agreed well with clinical findings in laterality.

## 1 Introduction

MSA is a sporadic, progressive, neurodegenerative disorder clinically characterized by autonomic dysfunction, parkinsonism, cerebellar ataxia, and pyramidal signs (Quinn, 1989). MSA can be classified into two subgroups, a cerebellar (MSA-C) and a parkinsonian (MSA-P) variant (Gilman et al., 2008). Neuropathologically, MSA is characterized by selective neuronal loss and reactive gliosis predominantly affecting the basal ganglia, substantia nigra, olivopontocerebellar pathways and the intermediolateral cell column of the spinal cord (Papp and Lantos, 1994; Wenning et al., 1997). The histological hallmarks of MSA are α-synuclein-positive glial cytoplasmic inclusions in the oligodendroglia, which are required for the diagnosis of definite MSA (Gilman et al., 2008; Papp and Lantos, 1994; Wenning et al., 1997).

VBM is a method of statistically analyzing morphological changes in the brain as measured by whole-brain MRI data (Ashburner and Friston, 2000). In the past few years, VBM has been used to study the patterns of structural changes in the brain during brain development or in neurodegenerative disorders (Bergfield et al., 2010; Brenneis et al., 2004; Burton et al., 2002). In MSA-P, VBM revealed gray matter loss mainly in the striatum, the cerebral cortex including the motor area and the cerebellar lobes (Brenneis et al., 2003, 2007; Minnerop et al., 2007; Tir et al., 2009; Tzarouchi et al., 2010). However, white matter volume changes have been rarely reported, and the results were inconsistent (Brenneis et al., 2003; Minnerop et al., 2007, 2010; Tzarouchi et al., 2010).

In the present study, we evaluated MR images of MSA-P patients to examine the volume changes of white matter as well as gray matter by using the latest VBM technique with SPM 8 plus DARTEL (Matsuda et al., 2012).

## 2 Materials and methods

### 2.1 Participants

We retrospectively reviewed an electronic database of radiology reports for 12,029 patients who underwent brain MRI examinations at our institution between March 2007 and September 2010 and searched for reports that indicated Parkinson's disease and related disorders. After 127 patients were indicated by the radiological reports, medical records revealed 23 patients who were diagnosed as possible or probable MSA-P according to consensus criteria (Gilman et al., 2008). Among 23 patients, three patients were excluded because of the presence of multiple lacunar infarctions in two patients and multiple cavernous hemangiomas in one patient. All volumetric T1-weighted images were visually inspected for apparent artifacts due to patient motion or metallic dental prostheses. As a consequence, 20 patients (7 men and 13 women; age range 48–77 years, mean age 62.9 ± 7.7 years, disease duration 4.1 ± 2.2 years) were enrolled as subjects in this study. The patient data are given in Table 1. Among these patients, 14 patients had left-side dominant (5 men and 9 women; age range 53–77 years, mean age 64.1 ± 6.4 years, disease duration 4.2 ± 2.5 years) and 6 patients had right-side dominant onset symptoms (2 men and 4 women; age range 48–73 years, mean age 59.8 ± 10.0 years, disease duration 3.7 ± 1.2 years). As a measure of disease severity, we adopted the following disease stages previously described: stage 0 = no gait difficulties, stage 1 = disease onset, as defined by onset of gait difficulties, stage 2 = loss of independent gait, as defined by permanent use of a walking aid or reliance on a supporting arm, stage 3 = confinement to wheelchair, as defined by permanent use of a wheelchair (Klockgether et al., 1998).

Our local ethics committee did not require approval or patient informed consent for the retrospective review. Thirty age-matched control subjects (10 men and 20 women; age range 48–80 years, mean age 64.7 ± 7.7 years) were also involved as healthy control subjects.

Thirty age-matched healthy controls (10 men and 20 women; age range 48–80 years, mean age 64.7 ± 7.7 years) were also involved as control subjects. None had a history of neurological or psychiatric illness, and no abnormalities were observed on their brain structural MRIs. Institutional review board approval and written informed consent were obtained from the control subjects.

### 2.2 Image acquisition and analysis

All examinations were performed with a 1.5 T MR imaging system (Symphony Vision; Siemens, Erlangen, Germany). MR protocol for the parkinsonian is as follows. High-resolution three-dimensional (3D) T1-weighted images were acquired using magnetization-prepared rapid acquisition of a gradient echo sequence (144 sagittal sections, TR = 1600 ms, TE = 2.6 ms, flip angle = 15°, voxel size = 1.2 × 1.2 × 1.2 mm^3^, FOV = 315 mm, matrix = 208 × 256, 1.2-mm thickness with no gap). Axial T2-weighted images (TR = 3800 ms, TE = 95 ms, flip angle = 150°, voxel size = 0.7 × 0.4 × 5.0 mm^3^, FOV = 230 mm, matrix = 281 × 512, 5-mm thickness with 1.8-mm gap) and coronal fluid attenuation inversion recovery images (TR = 9000 ms, TE = 100 ms, flip angle = 170°, voxel size = 1.2 × 0.9 × 5.0 mm^3^, FOV 230 mm, matrix = 192 × 256, 5.0-mm thickness with 1.8-mm gap) were also obtained.

Using the latest version of SPM8 (Wellcome Department of Imaging Neuroscience, London, United Kingdom), we segmented the MRIs into gray matter, white matter, and cerebrospinal fluid images by a unified tissue-segmentation procedure after image-intensity nonuniformity correction. These segmented gray and white matter images were then spatially normalized to the customized template in the standardized anatomic space by using DARTEL (Wellcome Department of Imaging Neuroscience) (Ashburner, 2007). To preserve the gray and white matter volumes within each voxel, we modulated the images using the Jacobean determinants derived from the spatial normalization by DARTEL and then smoothed them using an 8-mm FWHM Gaussian kernel.

Morphological group differences for these smoothed gray and white matter images between all of the MSA-P patients and the controls were analyzed using a 2-sample *t*-test in SPM8. The same analysis was performed between the 14 left-side dominant onset MSA-P patients and the controls and between the 6 right-side dominant onset MSA-P patients and the controls. Group comparisons by SPM8 were assessed using the false discovery rate at a threshold of p < .05, corrected for multiple comparisons.

Additionally, for the correlation analyses with disease duration and disease stage, we used a multiple regression analysis and an uncorrected threshold of p < .001.

## 3 Results

**Fig. 1:**
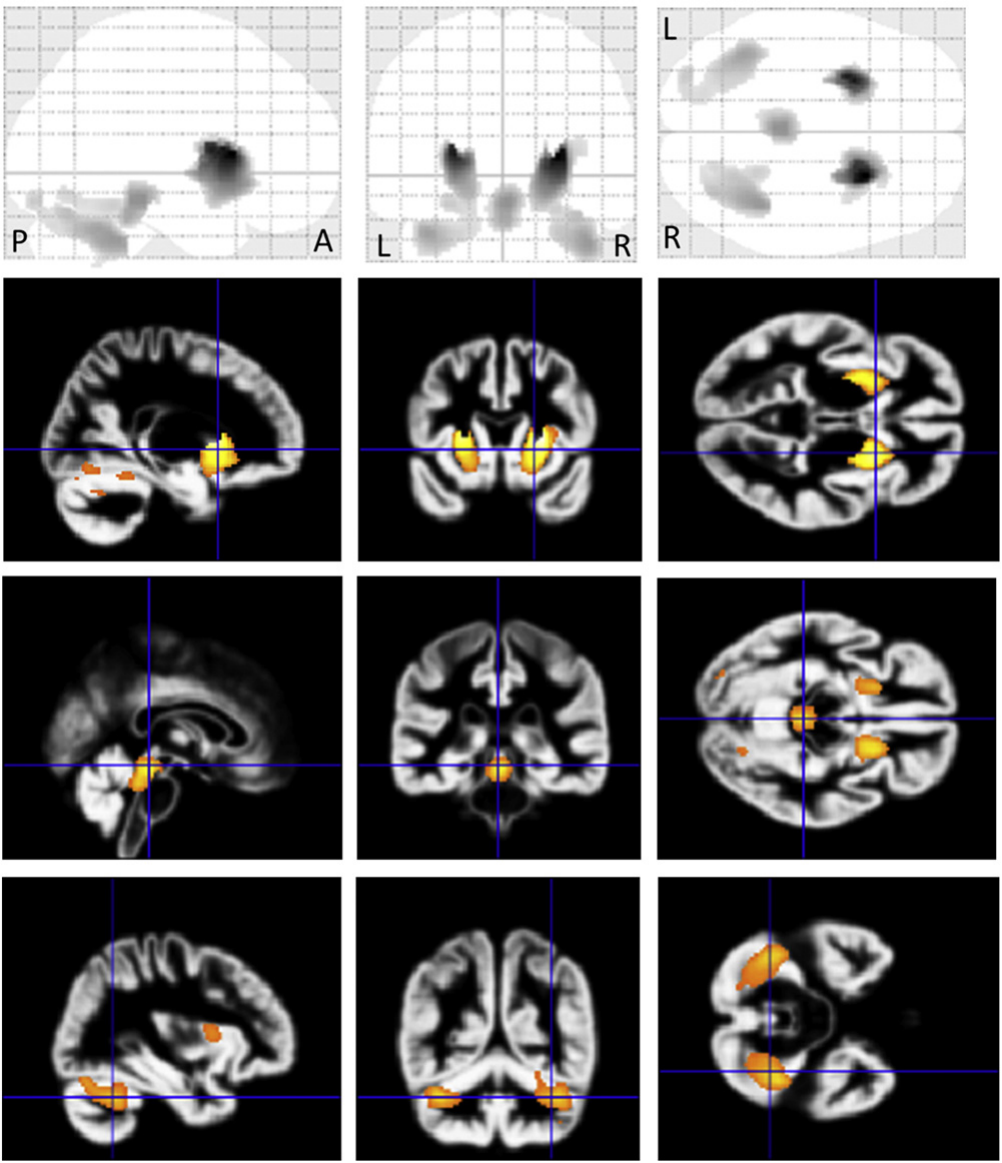
Comparison of gray matter volume by VBM using SPM8 plus DARTEL among 20 patients with MSA-P and 30 control subjects. Significant atrophy is observed in the bilateral putamina, cerebellums, dorsal midbrain and left inferior occipital gyrus in MSA-P patients compared to controls. Results are superimposed on the customized gray matter template (FDR-corrected at p < .05).

**Fig. 2:**
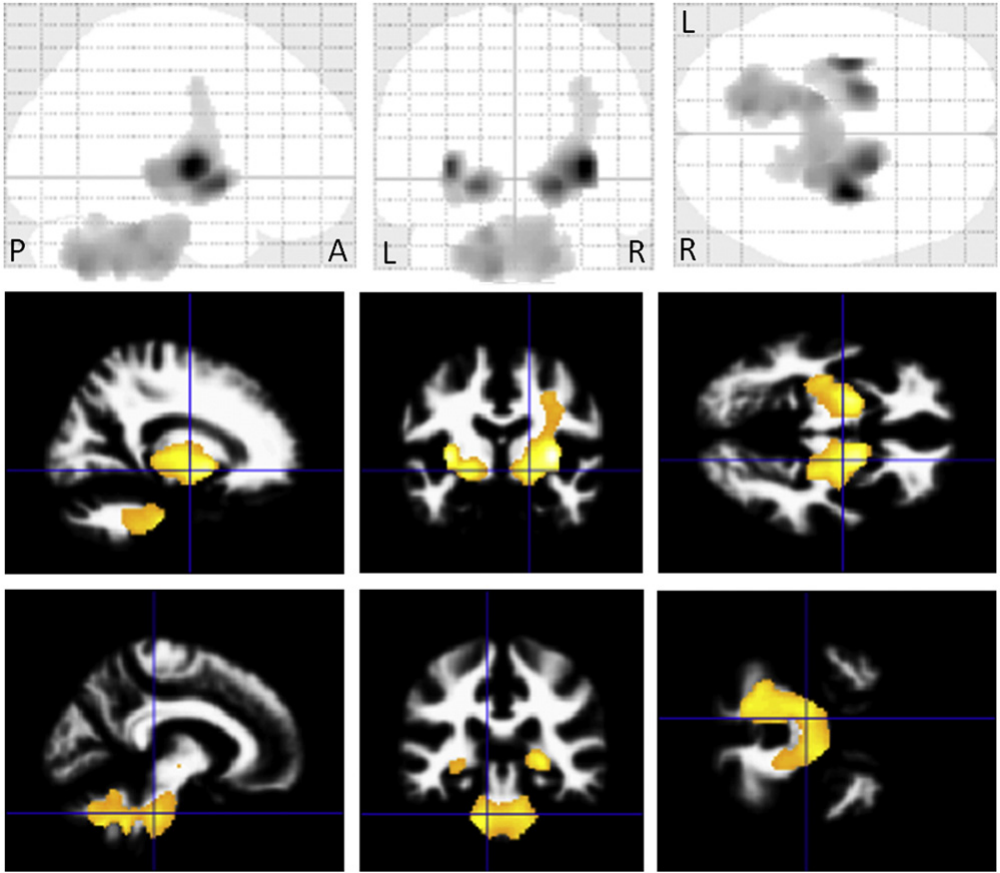
Comparison of white matter volume by VBM using SPM8 plus DARTEL among 20 patients with MSA-P and 30 control subjects. Significant atrophy is observed in the bilateral globus pallidi and external capsules extending to the midbrain. On the right side, the atrophy extends upward to the subcortical to precentral area through the internal capsule. White matter atrophy in the pons, bilateral middle cerebellar peduncles and left cerebellum is also detected. Results are superimposed on the customized white matter template (FDR-corrected at p < .05).

**Fig. 3:**
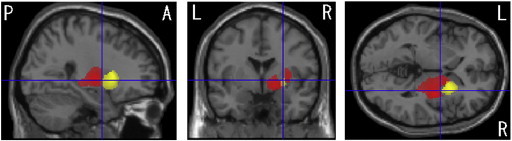
Comparison of gray and white matter volumes by VBM using SPM8 plus DARTEL among 14 patients with left-side dominant onset MSA-P and 30 control subjects. Significant gray matter atrophy which is shown in a yellow color is observed only on the right side of the putamen (FDR-corrected at p < .05). Significant white matter atrophy which is shown in a red color is observed only on the right side of the globus pallidus and external capsule (FDR-corrected at p < .05).

**Fig. 4:**
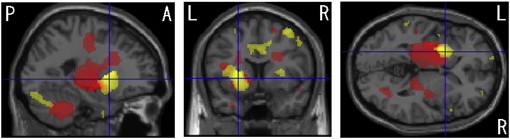
Comparison of gray and white matter volumes by VBM using SPM8 plus DARTEL among 6 patients with right-side dominant onset MSA-P and 30 control subjects. Significant gray matter atrophy which is shown in a yellow color is observed in the left putamen, bilateral cerebellums and several cortical regions (FDR-corrected at p < .05). Bilateral cerebellums and several cortical regions are partly shown. Significant white matter atrophy which is shown in a red color is observed in the left globus pallidus, bilateral external capsules, right frontal lobe, parahippocampal area and cerebellum (FDR-corrected at p < .05).

In MSA-P patients, VBM revealed regions of gray matter loss bilaterally affecting the putamina, cerebellums, dorsal midbrain and left inferior occipital gyrus (see Table 2, Fig. 1). Reduced white matter volume was located in the bilateral globus pallidi and external capsules extending to the midbrain (see Table 3, Fig. 2). On the right side, it extended upward to the subcortical to precentral area through the internal capsule. White matter loss in the pons, bilateral middle cerebellar peduncles and left cerebellum was also detected. In left-side dominant MSA-P patients, the putaminal gray matter was decreased only on the right side (see Table 4, Fig. 3). The reduced white matter was located in the right globus pallidus and external capsule (see Table 5, Fig. 3). In right-side dominant MSA-P patients, gray matter was reduced in the left putamen, bilateral cerebellums and several cortical regions (see Table 6, Fig. 4). The reduced white matter was located in the left globus pallidus, bilateral external capsules, right frontal lobe, right parahippocampal area and right cerebellum (see Table 7, Fig. 4). A correlation with disease duration and severity was not detected.

## 4 Discussion

To our knowledge, this is the first study to focus on the white matter volume loss in MSA-P patients as determined by VBM using SPM8 plus DARTEL. This analysis showed white matter atrophy in the globus pallidi and external capsules bilaterally extending to the midbrain. The white matter atrophy also spreads upward to the subcortical to right premotor area. These areas correspond to the regions connecting the pathologically affected structures. Such findings, which seemed to reflect the degeneration of the motor pathway, have never been presented in previous VBM studies. We believe that the evaluation of white matter as well as deep gray matter has significantly improved owing to this new software.

Neuropathological studies have shown neuronal loss and reactive gliosis in the putamen, caudate nucleus, external pallidum, substantia nigra, locus coeruleus, inferior olives, pontine nuclei, cerebellar lobes and intermediolateral cell columns of the spinal cord in MSA-P (Wenning et al., 1997). Most severe neuronal loss was found in the lateral part of the substantia nigra and dorsolateral putamen (Jellinger et al., 2005; Ozawa et al., 2004; Wenning et al., 2002). The previous VBM studies have reported gray matter loss in the putamen, caudate nucleus, cerebellar vermis and lobes, dorsal midbrain, and several cortical regions including the insular cortex and motor area (Brenneis et al., 2003, 2007; Chang et al., 2009; Minnerop et al., 2007, 2010; Tir et al., 2009; Tzarouchi et al., 2010). The significant putaminal loss detected in our study confirmed the findings of previous region of interest based morphometric and VBM studies (Brenneis et al., 2003, 2007; Minnerop et al., 2007; Schulz et al., 1999; Tzarouchi et al., 2010). Our VBM results also agree with the pathological features (Jellinger et al., 2005; Ozawa et al., 2004; Wenning et al., 1997, 2002).

Though some previous VBM studies have detected the atrophy of the caudate nucleus (Brenneis et al., 2003; Chang et al., 2009; Tzarouchi et al., 2010), our study did not detect volume loss in the caudate nucleus. Pathologically, the caudate nucleus is less involved than the putamen and tends to be relatively preserved in the early stage of MSA-P (Ozawa et al., 2004; Wenning et al., 2002). (Chang et al., 2009) reported that the caudate nucleus had significant atrophy compared to the putamen, a finding that is inconsistent with pathological features mentioned above. It is possible that the localization of deep gray matter at the periventricular space made it difficult to segment the MRIs in older versions of SPM. The recent report of Messina et al. (2011) supported our finding of no significant volume loss in the caudate nucleus as measured automatically by FreeSurfer.

Gray matter volume loss in the olivopontocerebellar system can also be seen in MSA-P, although the degree of involvement is lower than in MSA-C (Wenning et al., 1996, 1997). Atrophy of the cerebellar vermis and lobes was detected, which is in line with previous VBM studies (Chang et al., 2009; Minnerop et al., 2010; Tzarouchi et al., 2010) and pathological findings (Wenning et al., 1997). White matter cerebellar atrophy was also detected in addition to gray matter atrophy. The pons, bilateral middle cerebellar peduncles and the left cerebellar lobe were involved, a finding that was also consistent with previous VBM studies (Minnerop et al., 2010; Tzarouchi et al., 2010).

The white matter loss was detected in bilateral globus pallidi and external capsules extending to the midbrain which has never been reported before in a study using VBM. The white matter volume loss of the globus pallidus might reflect the fact that the globus pallidus has many efferent fibers and many bundles of myelinated fibers from the striatum traverse globus pallidus (Mamata et al., 2002; Nieuwenhuys et al., 2008). It is supported by the fact that the glucose metabolism of the globus pallidus is generally almost equal to white matter metabolism.

The areas connecting the globus pallidus with each structure were also significantly atrophic in this study. The neuronal loss in globus pallidus is pathologically proven (Jellinger et al., 2005; Ozawa et al., 2004; Wenning et al., 1997, 2002) and it plays an important role in parkinsonism as well as substantia nigra. Thus, those findings could result from the degeneration of the motor pathway such as striopallidal fiber, strionigral fiber and pallidonigral fiber (Nieuwenhuys et al., 2008). On the right side, the white matter volume loss extended upward to the subcortical to precentral area. The involvement of the precentral area could be explained by the fact that the motor cortex, the supplementary motor cortex and the premotor area were also involved in the pathways mentioned above; the afferent fibers of the striatum originate mainly from the motor and premotor areas, and then go back to the area (Nieuwenhuys et al., 2008). Another possibility is that the corticospinal tract itself is affected by this disease. The recent VBM studies of Minnerop et al. (2010) revealed white matter reduction along the corticospinal tract in the bilateral internal capsules and subcortical to left precentral gyrus, findings that agree with our study. However, they did not detect white matter atrophy around the deep gray matter. We believe that this was because they evaluated MSA patients including both MSA-C and MSA-P patients, and MSA-C patients made up more than 70% of their patient groups.

This better detection of deep gray matter and white matter volume loss in our study probably resulted from the application of the SPM8 plus DARTEL algorithm (Matsuda et al., 2012). The latest version of SPM8 enabled more accurate segmentation of the MRIs into gray matter, white matter, and cerebrospinal fluid images compared to older versions of SPM. In addition, DARTEL provides improved registration accuracy compared with conventional VBM.

In this study, we also evaluated both 14 left-side dominant and 6 right-side dominant onset MSA-P patients and detected the contralateral putaminal atrophy. Our results agreed with the previous MR imaging and pathological findings (Fearnley and Lees, 1990; Kato et al., 1992; Kume et al., 1992). The reduced white matter was also predominant in the contralateral globus pallidus and external capsule extending to the level of the midbrain. To our knowledge, this is also the first report to mention the laterality of the clinical findings and of white matter volume loss using VBM. The detection of volume loss only on the right side in the corona radiate of 20 MSA-P patients might be due to the large number of left-side dominant patients.

This study has several limitations. First, the number of patients in this study was not large. This might be the main reason for the failure of detecting the correlation with disease duration and severity. A few previous VBM studies with a small number of patients have reported inconsistent results on correlation with disease duration and severity (Brenneis et al., 2007; Minnerop et al., 2007, 2010). A further study with a large number of MSA-P patients would be necessary. Second, this study lacked the pathologic confirmation. However, it was difficult to select data only for pathologically confirmed patients, and the diagnosis in this study was clinically evaluated by an experienced neurologist based on consensus criteria. Third, we failed to detect the gray matter volume loss of the substantia nigra as well as in previous VBM studies, although the neuronal loss of substantia nigra is a hallmark of MSA-P (Mamata et al., 2002; Minnerop et al., 2010; Tir et al., 2009). Even though the software used for the analysis and the evaluation of spatial resolution have greatly improved, the segmentation of MRIs of nuclei located in brainstem remains a weakness.

## 5 Conclusions

In conclusion, VBM using SPM8 plus DARTEL detected significant volume loss not only in the gray but also in the white matter of the area affected by MSA-P. Significant structural atrophic change of the areas connecting the globus pallidus with each structure which plays a crucial role in parkinsonism was detected for the first time using VBM. Further prospective investigations involving a larger number of MSA-P patients combined with DTI techniques are required to confirm our findings. VBM using SPM8 plus DARTEL could also be a useful tool for evaluating other neurodegenerative diseases as well as MSA-P.

## Figures and Tables

**Table 1:** Demographic characteristics of MSA-P patients and controls.

Characteristic	MSA-P	Controls
Age (y)	62.9 ± 7.7 (48–77)	62.9 ± 7.7 (48–80)
Sex	7 men, 13 women	10 men, 20 women
Diagnosis	16 probable MSA-P, 4 possible MSA-P	
Disease duration (y)	4.1 ± 2.2 (2–10)	
Stage 1	6	
Stage 2	11	
Stage 3	3	
Cerebellar symptoms	7 present, 13 absent	
Pyramidal signs	7 present, 13 absent	
Urinary incontinence	15 present, 5 absent	
Orthostatic hypotension	13 present, 7 absent	

Note: Unless otherwise indicated, data are means ± standard deviations, with ranges in parentheses. Stage 0 = no gait difficulties, stage 1 = disease onset, as defined by onset of gait difficulties, stage 2 = loss of independent gait, as defined by permanent use of a walking aid or reliance on a supporting arm, stage 3 = confinement to wheelchair, as defined by permanent use of a wheelchair.

**Table 2:** Clusters of gray matter loss (20 MSA-P vs. 30 controls).

Region volume (mm^3^)	Z score	Talairach coordinates (x, y, z)	Location of local maxima
9680	4.46	40, − 46, − 36	Right cerebellar tonsil
	3.79	36, − 69, − 15	Right cerebellum
	3.55	24, − 46, − 13	Right cerebellum
8424	6.36	26, 14, 7	Right putamen
6960	4.31	− 40, − 54, − 23	Left cerebellum
	3.6	− 28, − 84, − 9	Left inferior occipital gyrus
	3.31	− 20, − 75, − 20	Left cerebellum
5712	6.05	− 26, 8, 7	Left putamen
3896	4.6	0, − 34, − 12	Dorsal midbrain

Voxel size 2 × 2 × 2 mm^3^. Clusters of gray matter SPM analysis with FDR-corrected at p < .05 are shown. The coordinates refer to the Talairach reference space.

**Table 3:** Clusters of white matter loss (20 MSA-P vs. 30 controls).

Region volume (mm^3^)	Z score	Talairach coordinates (x, y, z)	Location of local maxima
32,144	4.23	− 12, − 64, − 34	Left cerebellum
	4.12	− 16, − 27, − 32	Left pons
	4.07	− 16, − 38, − 22	Left cerebellum
20,360	5.46	32, − 10, 2	Right external capsule
	4.94	18, 2, − 5	Right lateral globus pallidus
	3.35	36, − 6, 32	Right sub-precentral area
10,272	5.04	− 34, − 8, 4	Left external capsule
	4.79	− 20, − 2, − 5	Left lateral globus pallidus

Voxel size 2 × 2 × 2 mm^3^. Clusters of gray matter SPM analysis with FDR-corrected at p < .05 are shown. The coordinates refer to the Talairach reference space.

**Table 4:** Clusters of gray matter loss (14 left-side dominant onset MSA-P vs. 30 controls).

Region volume (mm^3^)	Z score	Talairach coordinates (x, y, z)	Location of local maxima
6864	6.38	26, 14, 5	Right putamen

Voxel size 2 × 2 × 2 mm^3^. Clusters of gray matter SPM analysis with FDR-corrected at p < .05 are shown. The coordinates refer to the Talairach reference space.

**Table 5:** Clusters of white matter loss (14 left-side dominant onset MSA-P vs. 30 controls). Voxel size 2 × 2 × 2 mm^3^. Clusters of gray matter SPM analysis with FDR-corrected at p < .05 are shown. The coordinates refer to the Talairach reference space.

Region volume (mm^3^)	Z score	Talairach coordinates (x, y, z)	Location of local maxima
13,448	5.51	32, − 8, 4	Right external capsule
	4.93	18, 0, − 5	Right lateral globus pallidus

**Table 6:** Clusters of gray matter loss (6 right-side dominant onset MSA-P vs. 30 controls).

Region volume (mm^3^)	Z score	Talairach coordinates (x, y, z)	Location of local maxima
11,832	5.76	− 27, 6, 5	Left putamen
	3.76	− 61, − 5, 8	Left superior temporal gyrus
	3.70	− 36, − 9, 13	Left insula
11,216	4.84	12, − 7, 45	Right cingulate gyrus
	4.52	2, 17, 34	Right cingulate gyrus
	4.18	− 12, − 8, 43	Left cingulate gyrus
5536	3.76	20, − 70, − 8	Right lingual gyrus
	3.62	24, − 76, − 11	Right fusiform gyrus
	3.55	38, − 54, − 26	Right cerebellum
4504	3.86	− 18, − 86, − 11	Left fusiform gyrus
	3.50	− 36, − 58, − 24	Left cerebellum

Voxel size 2 × 2 × 2 mm^3^. Clusters of gray matter SPM analysis with FDR-corrected at p < .05 are shown. The coordinates refer to the Talairach reference space.

**Table 7:** Clusters of white matter loss (6 right-side dominant onset MSA-P vs. 30 controls).

Region volume (mm^3^)	Z score	Talairach coordinates (x, y, z)	Location of local maxima
101,384	6.05	− 34, − 10, 2	Left external capsule
	5.68	− 22, − 6, − 6	Left lateral globus pallidus
	4.79	22, − 49, − 19	Right cerebellum
4912	4.06	22, − 32, 0	Right external capsule
	3.88	34, − 12, 2	Right external capsule
	3.65	24, − 8, − 10	Right parahippocampal area
4288	4.08	30, 5, 26	Right frontal lobe
	3.97	46, 5, 20	Right frontal lobe
	3.71	28, − 2, 41	Right frontal lobe

Voxel size 2 × 2 × 2 mm^3^. Clusters of gray matter SPM analysis with FDR-corrected at p < .05 are shown. The coordinates refer to the Talairach reference space.
